# Recycling of Waste Facial Masks as a Construction Material, a Step towards Sustainability

**DOI:** 10.3390/ma15051810

**Published:** 2022-02-28

**Authors:** Maria Idrees, Arslan Akbar, Abdeliazim Mustafa Mohamed, Dina Fathi, Farhan Saeed

**Affiliations:** 1 Department of Architectural Engineering & Design, University of Engineering & Technology, Lahore 54000, Pakistan; mariaidrees@uet.edu.pk; 2 Department of Architecture and Civil Engineering, City University of Hong Kong, Kowloon 999077, Hong Kong, China; 3 Department of Civil Engineering, College of Engineering, Prince Sattam Bin Abdulaziz University, Alkharj 16273, Saudi Arabia; a.bilal@psau.edu.sa; 4 Building & Construction Technology Department, Bayan College of Science and Technology, Khartoum 210, Sudan; 5 Structural Engineering and Construction Management Department, Faculty of Engineering and Technology, Future University in Egypt, New Cairo 11845, Egypt; dina.mohamed@fue.edu.eg; 6 Department of Polymer Engineering, University of Engineering & Technology, Lahore 54000, Pakistan; f.saeed@uet.edu.pk

**Keywords:** waste management, face masks recycling, mechanical properties, durability, green concrete, circular economy

## Abstract

Amid the COVID-19 pandemic, a sudden surge in the production and utilization of disposable, single-use facial masks has been observed. Delinquency in proper disposal of used facial masks endangers the environment with a new form of non-biodegradable plastic waste that will take hundreds of years to break down. Therefore, there is an urgent need for the resourceful recycling of such waste in an environmentally friendly way. This study presents an efficient solution by using waste masks in fibered or crushed form to produce environmentally friendly and affordable green concrete. This investigation assessed the mechanical and durability properties of waste masks-incorporated concrete. A total of six mixes were prepared for standardized tests to determine compressive strength, split cylinder tensile strength and rapid chloride penetration test (RCPT), and freeze-thaw resistance. The percentage of mask fibers used were 0.5, 1, 1.5, and 2% of concrete by volume, while crushed masks were used at 0.5% only. The mask waste in both forms was found suitable to be used in concrete. One percent of waste mask fibers was found as an optimum value to increase compressive and tensile strength, reduce chloride permeability, and increase freeze-thaw resistance. Besides this, 0.5% crushed mask fiber also performed well, especially for producing less permeable and highly durable concrete. It is thus corroborated that waste masks that increase pollution worldwide can be utilized sustainably to help build green buildings. By reutilizing waste masks to produce improved concrete with better strengths and higher durability, circular economy and sustainability are achieved, along with efficient waste management.

## 1. Introduction

The consumption of personal protective equipment (PPE), including facial masks, has dramatically increased worldwide due to the COVID-19 pandemic. The increase in the use of COVID-19 sanitary masks has given rise to new environmental challenges by adding vast plastic particle waste to the environment [1,2]. Almost 6600 million masks, weighing 2640.79 tonnes, are utilized every day. Disposable face masks are manufactured using non-woven polypropylene fabric. Two different fabrics (i.e., spun-bond polypropylene and melt-blown polypropylene) are used as raw materials for surgical and non-surgical face masks [3,4]. Similarly, polyethylene, polyurethane, polyacrylonitrile, polyester, and cotton fibers are also utilized as raw materials [5,6]. Waste masks adulterate waterways, freshwater, and marine environment and add plastics to the aqua medium. They are commonly seen on streets and beaches due to unawareness and mismanagement. Several countries and the World Health Organization (WHO) have issued regulations and guidelines on the waste management of PPE and the disposal of plastic waste, which also includes facial masks [7].

Face masks are a direct source of microplastics and pollutants in the environment [6]. The disintegration of face masks in micro, and nano plastic waste due to various environmental factors (e.g., temperature, humidity, and salinity) deteriorates living creatures’ health and worsens the environmental situation [8,9]. A single disposable mask releases around 1.5 million microplastic particles by weathering actions [9]. Similarly, the improper disposal of face masks can spread the disease and negatively affect the environment [10,11]. The adsorption of organic and inorganic nutrients on plastic waste, especially in water, can provide a supportive environment for pathogenic species like bacteria and contaminants to propagate further [12,13,14].

Unlike biomedical wastes, waste masks mainly were disposed of in mixed methods and not treated as bio-medical waste [15]. Infectious waste collection and deposition in developing countries is a significant health risk [16]. The incineration method to dispose of waste masks is not highly recommended due to toxic gases (dioxin and furan) generated during the incineration of plastics.

Disposing of waste masks mindfully and avoiding environmental pollution is a new challenge for researchers [17,18]. The possible solution to these issues is recycling used sanitary masks and reutilizing them as reinforcement in construction materials [19]. It helps in reducing the waste produced by masks globally and has positive effects on various properties of concrete. Some researchers have incorporated masks with paper pulp and admixtures in concrete [20]. Rehman et al. used the waste mask in fat clay to improve its mechanical properties [21]. Researchers have also explored the utilization of crushed fiber in roads and pavement bases [22]. RMIT University researchers are working on this technology. Hamdani et al. also suggested the possible recycling of waste masks, including usage in concrete [23].

There exist critical environmental and socio-economic issues tied to an increase in mask waste. There is a need to find a valuable way to use this waste, a topic that has been the subject of considerable research across the globe. This investigation explores the possibility of utilization of waste mask fiber and shredded masks. This research primarily focuses on laboratory experimentation and incorporates waste mask fiber and shredded mask into concrete without compromising its fundamental mechanical and physical properties. The scope of this study was accomplished by carrying out extensive research work at different stages and conducting significant tests on fiber-reinforced concrete.

Fibers are added to concrete to increase its structural integrity. The properties of concrete vary with type, geometry, distribution, orientation, and density [24,25]. Fibers control cracking due to plastic shrinkage and increase shatter resistance. Fibers are commonly added to concrete as a percentage of concrete volume, typically ranging from 0.1 and 2% to obtain optimized results [24]. Higher percentages of fiber may decrease concrete strength because it is tough to distribute fibers evenly over that amount, causing interlocking of fibers [26,27].

Virgin polypropylene fibers decrease workability and increase tensile and flexural strength significantly, thus mitigating cracking due to the fiber’s bridging effect. However, compressive strength can be affected (short fiber may improve it). Durability and dimensional stability are improved. Specifically, shrinkage-related issues are controlled by using propylene fiber [28,29]. Freeze-thaw resistance increases by using polypropylene fiber [30], which reduces permeability and ingression of harmful ions in concrete and has better chloride resistance [31].

This research is a step forward in addressing the disposal problem of used sanitary masks, which is a severe environmental issue globally. Additionally, it explores the possibility of reutilizing this waste material by using it in concrete production. Moreover, the fiber used in concrete to enhance its properties is expensive. The production of virgin fibers, i.e., carbon fibers, is considered harmful to the environment due to significant energy consumption and carbon emissions. Recycling waste masks in fiber form provides a cheaper source of fibers as construction materials and may replace the existing virgin fibers. Thus, using these materials in concrete is a partial solution to environmental and ecological problems. Furthermore, their utilization in construction materials may improve the microstructure of mortar and concrete with consequent improvement in their mechanical and durability characteristics.

## 2. Materials

Ordinary Portland Cement conforming to ASTM-C150 was used in the investigation. Sand with a fineness modulus of 2.42 was used as fine aggregate and crushed stone as coarse aggregate. Potable water and polycarboxylic-based superplasticizer at 0.5% were also used. Three-ply waste masks were used in this study because people mostly use them as they are inexpensive. The inner and outer layer of the face mask is spun-bond polypropylene fabric, and the middle layer is primarily melt-blown polypropylene fabric. However, in some cases, spun-bond polypropylene may be used in all three layers to reduce costs. The inner layer (melt-blown polypropylene) is the primary material that protects against viruses and polluted particles. During the COVID-19 pandemic, there was a shortage of melt-blown material initially, and costs increased worldwide. The ear loop of the face mask is made of either polyester or nylon. The material used to produce face masks is water-resistant and thermal resistant due to a plastic base, showing less thermal conductivity. All these properties are desirable in construction materials.

## 3. Methodology

### 3.1. Waste Masks

#### 3.1.1. Waste Mask Treatment

The chances of getting COVID-19 are 100 times lesser from surfaces rather than acquiring from an infectious person directly [32,33]. Other studies show coronavirus can live up to three days on plastics and one day on cardboard [34]. Utilization of facemask in concrete is not problematic because the possibility of spread of COVID-19 through face masks diminishes because of the non-livability of the virus on plastics for a long time. However, as suggested by WHO, steam treatment can also be applied to disinfect the masks. As concrete has a pH of 13, the survival of the virus may also be challenging in such an environment.

The masks were collected and left for seven days. For further protection, they were disinfected by using an alcohol-based disinfectant spray.

**Figure 1:**
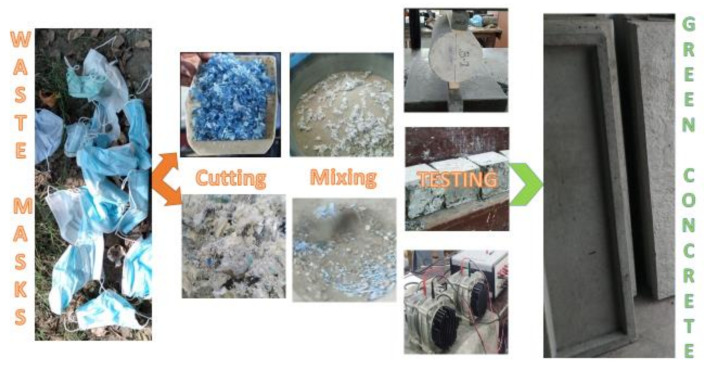
Methodology to use waste masks in concrete.

Researchers are currently exploring the possibility of utilizing waste PPE as an effective construction composite material [35]. Figure 1 shows the methodology followed in this study. The waste masks are processed into two forms, fibered and crushed in squares.

#### 3.1.2. Cutting of Masks

##### Fibered Form

Fibers are usually expensive; thus, using fibers extracted by waste will lead to sustainability and a circular economy. As fibers are required in concrete to enhance various properties, the masks were fibered in a machine that extracts fibers from cotton. The mask fibers were incorporated at 0.5, 1.0, 1.5, and 2.0% of concrete volume. Because the fibers were extracted from a cotton fiber extracting machine, they were not of uniform shape and size and resembled a rough, untidy cluster (see Figure 1).

##### Crushed Form

The masks were crushed in 1.5–2.0 mm squares using a paper cutting machine. This is the most readily and usable form of waste mask. Crushed masks were used as 0.5% of total concrete volume.

### 3.2. Mixing and Casting

Locally available coarse aggregates and river sand were used along with cement in concrete at the constant water-cement ratio of 0.5. The crushed fibers were incorporated at 0.5% of concrete volume, while the fibered masks were used at 0.5, 1.0, 1.5, and 2.0% of concrete volume. The mix proportion is tabulated in Table 1.

Cement, sand crush, and masks were first dry mixed for 1 min, and then superplasticizer and water were added. Mixing was continued for a further 2 min. After a 1 min break, mixing resumed again for two minutes. The slump of all mixes was found to be acceptable and within the range of 80 to 120 mm. Concrete was cast into 6 × 6 × 6 inches cubes and 4 × 8 inches cylinders for each batch. After 24 h of casting, the samples were demolded and placed in water at room temperature in a laboratory until the test day.

### 3.3. Mechanical Tests

Compressive strength tests and split cylinder tensile strength tests were conducted on samples after 28 days of curing. Compressive strength tests were performed on each batch’s six cubes (6 in × 6 in × 6 in) by applying a load of 0.25 MPa/s. Split cylinder tensile strength tests conforming to ASTMC496 were conducted on 4 × 8 inch cylinders, four cylinders per batch for six batches [36].

### 3.4. Durability Tests

The rapid chloride permeability test (RCPT) is a good indicator of permeability (interconnectivity of pores in concrete causing water and ions to ingress and attack). Higher permeability means more durability issues [37,38]. Higher values of RCPT provide higher chances of steel corrosion in concrete. Hence, the RCPT apparatus was used, conforming to ASTM C 1202 to obtain the permeability of different mixes.

The durability of concrete reduces in harsh environments [39]. Freeze-thaw is a common issue globally. Synthetic fibers increase freeze-thaw resistance [40]. Thus, the effect of waste mask fiber on the freeze-thaw resistance of concrete was also explored. For this purpose, small concrete cylindrical samples were exposed to freezing temperature for 6 hours and the next 6 hours at room temperature (30 to 40 °C), for ten days. Afterwards, their freeze-thaw resistance was compared using the RCPT test. The more deteriorated samples should have higher cracks and permeability.

### 3.5. FESEM and EDAX

Field emission scanning electron microscopy (FESEM) was used to understand the microstructure of the concrete. Additionally, EDAX was also conducted to characterize the concrete.

### 3.6. Measurement of Thermal Properties

A thermogravimetric analyzer (Shimadzu, TGA-50) was used to determine thermal properties. Around 7 to 10 mg of each sample was placed in an aluminum pan for measurement. The tests were performed from 25 °C to 600 °C temperature at a 10 °C/min ramp. The aluminum pan with concrete samples was placed in the furnace of the TGA. The samples were heated at the mentioned rate, and mass loss in milligrams was measured with the increase in temperature. The results obtained from the TGA were analyzed using Shimadzu thermal analyzer software.

## 4. Results and Discussion

This research program used waste masks (both in fibered and crushed forms) in concrete production. The effect of waste masks on the properties of concrete was investigated.

### 4.1. Compressive Strength

**Figure 2:**
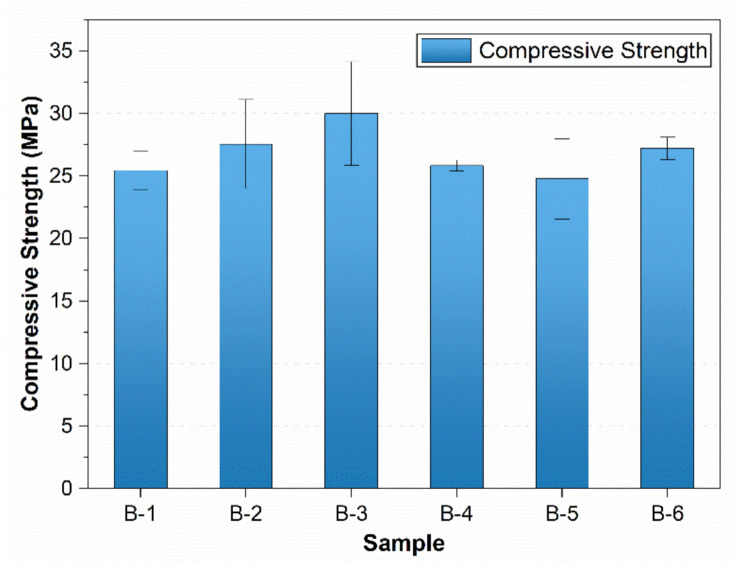
Compressive strength of waste mask-incorporated concrete at 28 days.

Figure 2 shows the compressive strength of the mixes at 28 days. The addition of 0.5% crushed fiber increased compressive strength by 8.3%. Compressive strength increased up to 1% fiber addition (by 17.9%) and then decreased. However, compressive strength remained higher than the control, up to 1.5%, and a minor decrease of 2% was observed by 2% fiber. The compressive strength decreased at a higher fiber volume due to improper mixing and interlocking of fiber, which does not allow homogeneity in the concrete [26]. Usually, compressive strength decreases by adding a higher amount of fiber. The property is attributed to the air entrapped during mechanical concrete mixing with a higher fiber content. An increase in polypropylene fiber content causes an increase in air content [25,41,42,43]. However, Guerini et al. tested polypropylene fiber at 0.5 and 1% by concrete volume and reported a meager increase in air content by increasing fiber content [44].

There are no entangling and conglobating of fibers and non-homogeneity issues with a lower amount of fibers [26,45]. At lower fiber volume, mixing is not improper. Hence, it does not cause much air entrapment and conglobating and thus increases strength. The conglobated fiber also causes bigger ITZ and lowers the strength.

Qin et al. studied the compressive strength of plain polypropylene fiber and waste polypropylene fabric fiber-incorporated concrete [46]. They found that waste fabric concrete increased compressive strength more than plain fiber because of the stress characteristics of fibers in the concrete, which offer better performance of waste mask fiber in concrete.

### 4.2. Split Tensile Strength Test

**Figure 3:**
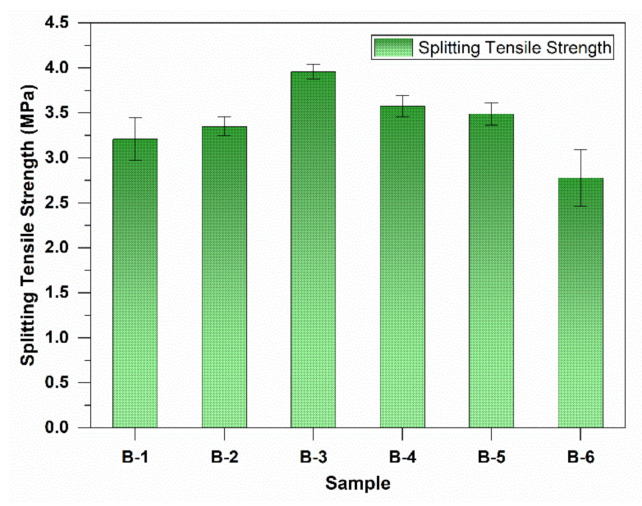
Splitting tensile strength of waste mask-incorporated concrete at 28 days.

Tensile strength is higher for mask fiber-added concrete up to 2% mask incorporation. However, tensile strength increased up to 1% fiber (by 23.3%) and then decreased, as shown in Figure 3. Mask fiber-added concrete can have a better tensile capacity and thus better cracking resistance than ordinary concrete. Garg and Garg found similar 28-day tensile strength [47]. They found that the tensile strength of polypropylene fiber at 0.8., 1.2, and 1.6% fiber volume was 32, 34, and 32% higher than the control; the trend is similar, showing maximum result with close to 1% fiber content.

However, the tensile strength for crushed fiber decreased by 13.4% because the mask square piece of 1.5 to 2 mm length did not contribute to the sample’s integrity against tensile strength. Mask pieces acted as a barrier and allowed the sample to split easily during the tensile strength test.

### 4.3. Rapid Chloride Permeability Test (RCPT)

**Figure 4:**
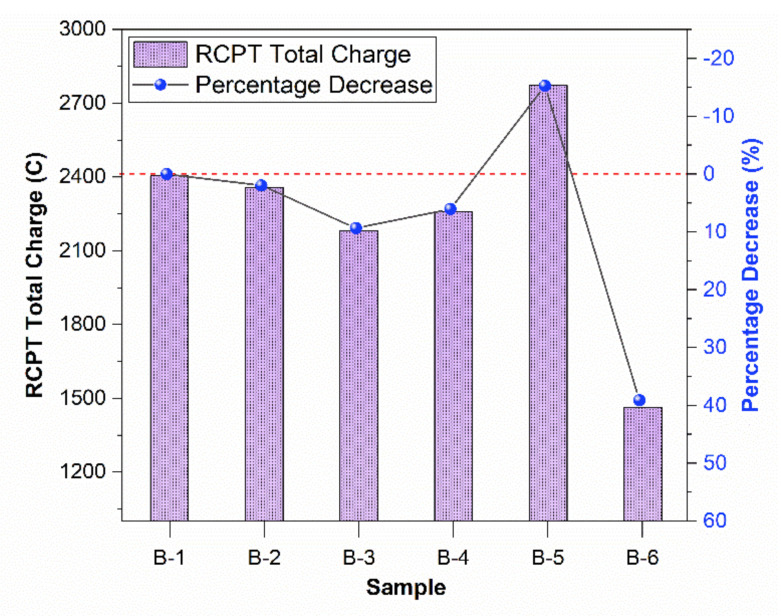
RCPT values of different concrete samples at 28 days.

The samples were checked for chloride permeability. Lower chloride permeability represents better corrosion resistance. Figure 4 shows that the permeability decreased first by adding waste mask fiber in concrete, up to 1% fiber. However, after 1% fiber, the permeability increases. It can be inferred that 1% is the optimum value at which permeability is decreased by 9.4%. At lower percentages, polypropylene fiber reduces permeability and increases the resistance against chloride penetration by filling the effect in pores [48].

However, after 1%, fiber causes air entrainment and increases permeability. A higher volume of polypropylene fibers causes a higher air content [49]. Improper mixing of fiber and non-homogeneity of mix may also cause air void content. At 2% fiber, the permeability increases due to higher air entrapped by fiber.

The trend can explain the compressive strength pattern, which increases up to 1% due to low air entrainment. At 1.5 and 2%, a higher amount of air is entrapped during mechanical mixing, thus reducing compressive strength.

The 39.1% decrease in permeability is visible for the crushed mask. The masks are impermeable, and the square-cut pieces do not allow water or ions to pass through them and cause a considerable decrease in the permeability of the concrete sample. Thus, 0.5% 1.5 mm square cut samples are excellent for use when permeability is the main criterion to be decreased.

### 4.4. Rapid Chloride Permeability Test (RCPT) of Freeze–Thawed Samples

It is evident that samples deteriorated and cracked after passing through freeze-thaw cycles. The cracks developed caused an increase in chloride permeability. The test applied on samples exposed to freeze-thaw cycles corroborated the fact that 1% fiber showed better resistance against freeze-thaw deterioration.

When water freezes in concrete, it expands in volume. As concrete cannot withstand tensile stresses caused by the expansion of water, cracks appear. The fiber can improve tensile strength and thus provide better resistance against cracks due to its crack-bridging property. Polypropylene fiber has an inhibiting effect on crack initiation and propagation [50].

**Figure 5:**
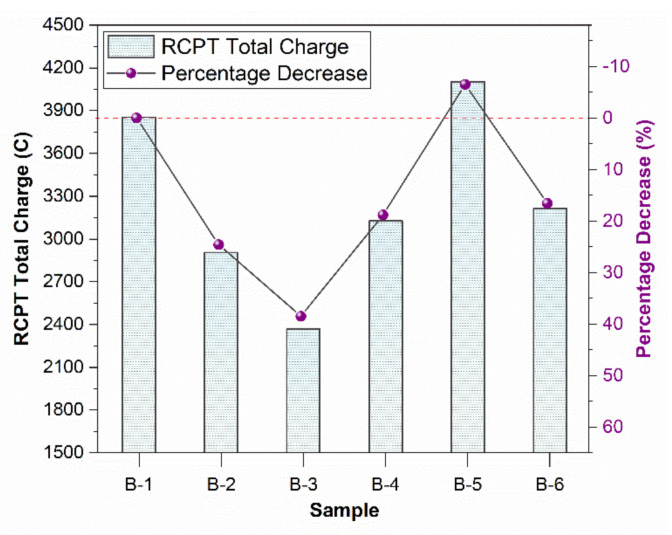
RCPT value of charge after freeze-thaw cycles.

Figure 5 shows the RCPT results of samples after exposure to freeze-thaw cycles. A value of 0.5% crush performed better than the control sample and showed 16.6% lower permeability. Additionally, 0.5% to 1.5% fiber provided better resistance than the control samples and 1% fiber showed 38.6% reduced permeability.

Waste mask usage in concrete provides better results than ordinary concrete against freeze-thaw attacks. Nam et al. found that synthetic fibers, especially PVA, are effective against frost resistance due to their air void content [51]. Evenly dispersed air voids may increase frost resistance by allowing freezing water to penetrate and swell within voids. Thus, the expansion of ice within the void does not exert pressure on concrete, thus leading to better frost resistance.

Using one percent fibered waste mask improved the flexural behavior mechanical properties of concrete (increase in split tensile strength and compressive strength). Additionally, it decreased permeability, as shown by RCPT results. Hence, using a one percent fibered waste mask provided the optimum solution for using waste masks in concrete.

### 4.5. FESEM and EDAX

**Figure 6:**
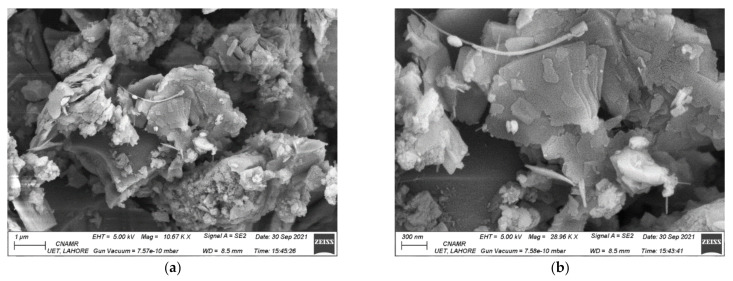
(**a**) FESEM at 1 µm, (**b**) FESEM at 300 nm.

FESEM images show the internal structure of concrete at 1% fiber content (optimum value for strength and durability). It was observed that polypropylene fibers are wrapped mostly by hydration products and are not very visible. The abnormal rectangular growths in the image represent overlapping/wrapping of CSH on the fiber of the waste masks. The cotton-like appearance in Figure 6a shows that fiber cotton has been wrapped mainly through CSH. However, the hydration products start to move around longer fibers (see Figure 6a,b). Because masks are made up of spun-bound polypropylene and melt-brown polypropylene layers, they may show different behavior as sites for hydration products. WHO guidelines also encourage that mask layers be made of hydrophilic (near the mouth) and hydrophobic layers of polypropylene and other materials [7].

Furthermore, it is evident from the EDAX results that CSH layers the surfaces of the fibers. The major content is Ca from cement and Si from silica sand and cement. The results show that all the fiber content in the concrete is well dispersed and completely hydrated by the CSH.

**Figure 7:**
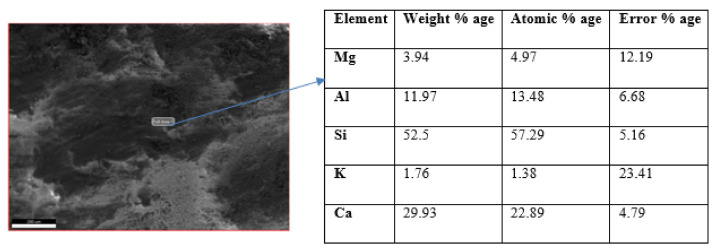
EDAX of concrete with its elemental analysis.

Figure 7 shows the Energy Dispersive X-ray Analysis (EDAX) spectrum of the 1% fibered mask concrete. The peaks in this spectrum show the presence of elements in the specimen. The high spikes of Ca and Si show C-S-H’s formation in the concrete. EDAX shows the chemical characterization of the sample. As no mineral admixture was added and polypropylene fiber does not alter the characterization, the characterization shows plain concrete. Fibers change the properties of concrete because of their fiber effect, not due to changes in chemical composition.

### 4.6. Thermal Analysis

**Figure 8:**
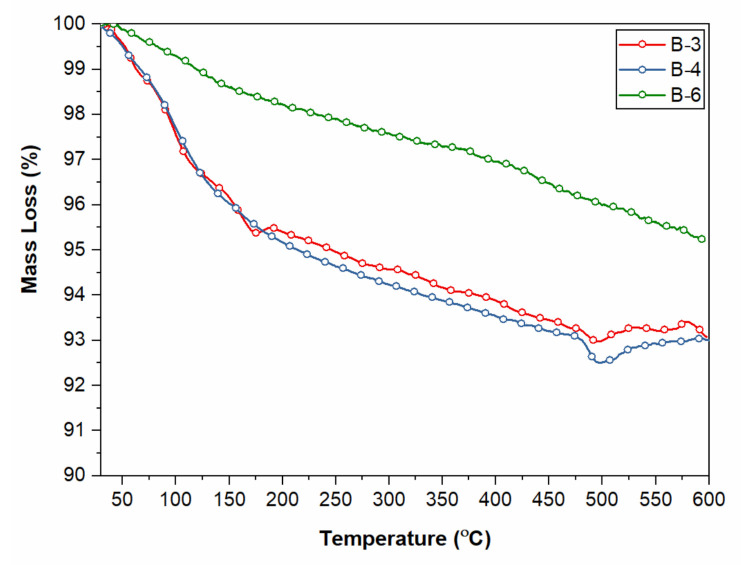
TGA analysis of waste mask-filled concrete.

The concrete with 0.5%, 1%, and 1.5% fibers from waste masks are tested for thermal degradation using TGA. The results show (Figure 8) that there is no significant degradation peak of polypropylene in the concrete. This might be due to the low percentage of the polypropylene fabric used in the concrete and does not provide a significant transition. Normally, polypropylene starts degrading at 320 °C, whereas the DTG curve represents the degradation temperature of polypropylene at 420 °C. The mass loss from 50 °C to 400 °C is due to drying of capillary pore residual water, and from 420 °C to 500 °C due to the dehydroxylation of calcium hydroxide of concrete [52,53]. The concrete with a 1.0% waste mask fiber shows slightly high mass loss due to capillary pore residual water drying. More drying means more water retention during curing of the concrete, and hence it shows better overall performance. There is no significant degradation of polypropylene fiber at high temperature, which signifies that the concrete will not produce any gas at high temperature and hence is safe at a higher temperature.

## 5. Conclusions

The following conclusions were corroborated by the investigation to find the feasibility of using waste facial masks as a construction material:

(1) The properties of concrete were affected by adding crushed and fibered masks. The addition of 0.5% crushed mask increased compressive strength by 8.3% but decreased tensile strength by 13.4%.
(2) Compressive and tensile strength of concrete increased with up to 1% fiber addition (by 17.9% and 23.3%) and then decreased. Thus, utilization of mask fibers at 1% of concrete volume was an optimum percentage to enhance mechanical properties.
(3) One percent waste mask fiber showed low chloride permeability, and crushed mask showed very low permeability than ordinary concrete. Hence, the corrosion resistance of concrete with waste masks, especially in crushed form, is relatively higher. Similarly, the rapid chloride permeability test showed a lower permeability value for mask-incorporated concrete after freeze-thaw cycles than ordinary concrete.
(4) Waste mask fiber at 2% showed slightly reduced compressive strength and high permeability and should not be used in concrete. Thus, the durability of concrete is improved when waste masks are added at 0–1.5% fiber content (1% optimum value).
(5) Crushed waste masks at 0.5% are also suitable for concrete, especially to improve water resistance.

Waste masks are produced, used, and wasted tremendously owing to the COVID-19 pandemic, and their dumping is a significant environmental issue. The addition of waste masks in concrete is an environmentally friendly solution. Recycling waste masks contributes to the circular economy. Further, it is a considerable step towards achieving sustainability of the environment with regard to waste-dumping issues, resource conservation, and environmental pollution reduction. Waste masks added to concrete in the form of fiber at 1% by volume of concrete is found to be the optimum percentage to enhance the mechanical and durability properties of concrete.

## Figures and Tables

**Table 1:** Mix proportions of concrete with and without fiber reinforcement.

Mixture ID	Ingredients						
	Cement (kg/m^3^)	Sand (kg/m^3^)	Coarse Aggregate (kg/m^3^)	Water (kg/m^3^)	Superplasticizer (%)	Fiber (%)	Remarks
B1	400	600	1200	200	0.5	-	-
B2	398	597	1194	199	0.5	0.50	Fibered
B3	398	597	1194	199	0.5	1.00	Fibered
B4	398	597	1194	199	0.5	1.50	Fibered
B5	398	597	1194	199	0.5	2.00	Fibered
B6	398	597	1194	199	0.5	0.50	Crushed

## Data Availability

The data presented in this study are available upon request from the corresponding author.
